# Editorial: The Neural Control of Locomotion: Current Knowledge and Future Research

**DOI:** 10.3389/fnhum.2022.950350

**Published:** 2022-06-20

**Authors:** Monika Pötter-Nerger, Ioannis U. Isaias

**Affiliations:** ^1^Department of Neurology, University Medical Center Hamburg-Eppendorf, Hamburg, Germany; ^2^Department of Neurology, University Hospital Würzburg, Würzburg, Germany; ^3^Parkinson Institute Milan, ASST G. Pini-CTO, Milan, Italy

**Keywords:** Parkinson's disease, gait disorder, freezing of gait, methodological approaches, virtual reality, therapeutical advances, electroencephalography, electromyography

Gait disturbances represent one of the most disabling symptoms in parkinsonian patients. In particular, freezing of gait is a peculiar gait derangement characterized by a sudden and episodic inability to produce effective stepping, causing falls, mobility restrictions, poor quality of life, and increased morbidity and mortality with high economic burden. Freezing of gait represents an enigmatic phenomenon and became the focus of intense basic and clinical research due to incomplete pathophysiological understanding and therapeutically limited options. This e-book, *The Neural Control of Locomotion: Current Knowledge and Future Research*, aims to collect scientific contributions regarding advances in the understanding and treatment of the Parkinsonian gait disorder. A total of sixteen papers with six original research manuscripts, eight reviews and two opinion papers have been included into this special issue to bridge pathophysiological knowledge from animal research to human gait studies covering three main topics. The first gathers different methodological approaches for a more accurate and standardized gait assessment such as gait analysis in fully immersive virtual reality environments, portable technologies, mobile electroencephalography, and the role of motor imagery. In the second section, research methods are integrated to illustrate complementary hypotheses on the pathophysiology of gait and gait freezing. This section begins with new hypotheses on freezing of gait as a generalized network phenomenon and a redefinition of the clinical symptomatology of freezing of gait. In addition to general considerations of locomotor network derangements in animal models and humans, specific aspects of locomotor control are discussed, such as the role of subpopulations of striatal neurons, the importance of low-frequency electromyographic activity of synergistic muscles and anticipatory postural adjustments during gait initiation. The third section bridges pathophysiological insights to actual and new therapeutic concepts. Beginning with a review of state-of-the-art medical concepts, novel rehabilitative strategies, such as repeated gait perturbation training, and new translational approaches using deep brain stimulation are discussed. Particularly, the benefits of deep brain stimulation with trouble-shooting options for gait are reviewed and new stimulation paradigms, e.g., combined subthalamic and nigral stimulation and lead symmetry are presented to improve gait control in parkinsonian patients. This e-book provides the opportunity to bridge the gap between basic neuroscience innovative therapeutic and rehabilitative concepts for further understanding and better treatment of parkinsonian gait disorder.

## Author Contributions

MP-N and II designed and conceptualized the content of this special issue Neural Control of Locomotion: Current Knowledge and Future Research and drafted this editorial introduction to the manuscript collection. All authors contributed to the article and approved the submitted version.

## Funding

This e-book was sponsored by the Deutsche Forschungsgemeinschaft (DFG, German Research Foundation) – Project-ID 424778381 – TRR 295 and the Fondazione Grigioni per il Morbo di Parkinson. Additionally this e-book was sponsored by DFG, German Research Foundation, SFB 936.

## Conflict of Interest

The authors declare that the research was conducted in the absence of any commercial or financial relationships that could be construed as a potential conflict of interest.

## Publisher's Note

All claims expressed in this article are solely those of the authors and do not necessarily represent those of their affiliated organizations, or those of the publisher, the editors and the reviewers. Any product that may be evaluated in this article, or claim that may be made by its manufacturer, is not guaranteed or endorsed by the publisher.

